# Incorporating an Intelligent Tutoring System Into a Game-Based Auditory Rehabilitation Training for Adult Cochlear Implant Recipients: Algorithm Development and Validation

**DOI:** 10.2196/55231

**Published:** 2024-12-03

**Authors:** Florian Gnadlinger, Maika Werminghaus, André Selmanagić, Tim Filla, Jutta G Richter, Simone Kriglstein, Thomas Klenzner

**Affiliations:** 1 Faculty of Informatics Masaryk University Brno Czech Republic; 2 University of Applied Sciences Berlin Berlin Germany; 3 Department of Otorhinolaryngology Hörzentrum University Hospital Düsseldorf Medical Faculty of Heinrich-Heine-University Düsseldorf Germany; 4 Clinic for Rheumatology University Hospital Düsseldorf Medical Faculty of Heinrich-Heine-University Düsseldorf Germany; 5 Hiller Research Center University Hospital Düsseldorf Medical Faculty of Heinrich-Heine-University Düsseldorf Germany; 6 AIT Austrian Institute of Technology GmbH Vienna Austria

**Keywords:** cochlear implant, eHealth, evidence-centered design, hearing rehabilitation, adaptive learning, intelligent tutoring system, game-based learning

## Abstract

**Background:**

Cochlear implants are implanted hearing devices; instead of amplifying sounds like common hearing aids, this technology delivers preprocessed sound information directly to the hearing (ie, auditory) nerves. After surgery and the first cochlear implant activation, patients must practice interpreting the new auditory sensations, especially for language comprehension. This rehabilitation process is accompanied by hearing therapy through face-to-face training with a therapist, self-directed training, and computer-based auditory training.

**Objective:**

In general, self-directed, computer-based auditory training tasks have already shown advantages. However, compliance of cochlear implant recipients is still a major factor, especially for self-directed training at home. Hence, we aimed to explore the combination of 2 techniques to enhance learner motivation in this context: adaptive learning (in the form of an intelligent tutoring system) and game-based learning (in the form of a serious game).

**Methods:**

Following the suggestions of the evidence-centered design framework, a domain analysis of hearing therapy was conducted, allowing us to partially describe human hearing skill as a probabilistic competence model (Bayesian network). We developed an algorithm that uses such a model to estimate the current competence level of a patient and create training recommendations. For training, our developed task system was based on 7 language comprehension task types that act as a blueprint for generating tasks of diverse difficulty automatically. To achieve this, 1053 audio assets with meta-information labels were created. We embedded the adaptive task system into a graphic novel–like mobile serious game. German-speaking cochlear implant recipients used the system during a feasibility study for 4 weeks.

**Results:**

The 23 adult participants (20 women; 3 men) fulfilled 2259 tasks. In total, 2004 (90.5%) tasks were solved correctly, and 255 (9.5%) tasks were solved incorrectly. A generalized additive model analysis of these tasks indicated that the system adapted to the estimated competency levels of the cochlear implant recipients more quickly in the beginning than at the end. Compared with a uniform distribution of all task types, the recommended task types differed (*χ*²_6_=86.713; *P*<.001), indicating that the system selected specific task types for each patient. This is underlined by the identified categories for the error proportions of the task types.

**Conclusions:**

This contribution demonstrates the feasibility of combining an intelligent tutoring system with a serious game in cochlear implant rehabilitation therapies. The findings presented here could lead to further advances in cochlear implant care and aural rehabilitation in general.

**Trial Registration:**

German Clinical Trials Register (DRKS) DRKS00022860; https://drks.de/search/en/trial/DRKS00022860

## Introduction

### Background

Globally, an estimated 1.5 billion people develop mild (<20-34 dB; approximately 1.1 billion people) to complete (>95 dB; approximately 12.6 million people) hearing loss [1,2]. Older adults (aged ≥50 years) are much more affected [1]. If traditional external sound-amplifying hearing aids cannot treat severe or profound (≥70 dB [3]) hearing loss anymore, a cochlear implant is a viable solution for specific individuals [4]. Implantation depends on the individual clinical picture—for example, for one (ie, single sided) or both (ie, bilateral) ears or together with an additional hearing aid (ie, bimodal) [5]—and other prerequisites, such as access to and financial support for aural rehabilitation [1,6,7]. The underlying idea of cochlear implants is to stimulate the auditory nerve from within the cochlea with electrical signals generated by an externally carried processor [1,4]. Hence, the damaged areas of the auditory system in the inner ear are bypassed through a cochlear implant, resulting in a coarser signal resolution than a normal hearing sensation [1,8]. Therefore, patients must learn and practice interpreting this new stimulus of the auditory nerve, especially for language comprehension [7]. Supporting this learning process, postsurgery auditory training is crucial for cochlear implant recipients [6,9,10].

Auditory training can be divided into 2 main categories: face-to-face auditory training guided by a therapist and self-directed, home-based auditory training by the cochlear implant recipients themselves [10]. For most cochlear implant recipients in many countries, ongoing face-to-face auditory training is unattainable owing to financial limitations and the unavailability of therapists [11]. Hence, cochlear implant recipients are usually supported with self-directed, home-based auditory training materials like reading aloud tasks; having someone else read specific content to the cochlear implant recipient; listening to audiobooks, the radio, or television; or computer-based auditory training [10].

Unlike other auditory training materials, computer-based auditory training provides benefits such as automated testing and scoring, progress monitoring, real-time corrective feedback, or customized training [6,10]. Therefore, computer-based auditory training (with a particular focus on cochlear implant recipients [6,10-14]) gained much attention as an inexpensive, low-threshold, and successful rehabilitation form [6,9,10]. However, Völter et al [14] noted that a crucial determinant for the success of self-directed auditory training lies in the intrinsic motivation of the patient to adhere to a given training. However, as the authors noted further, patients with chronic illnesses are often driven by external motivation [14]. The patients are aware that the learning and training will be exhausting or painful but observe it as a necessary step to reach a desirable and enjoyable outcome [15].

Drummond et al [15] argue that in the context of eHealth, serious games can specifically address extrinsically motivated learners if the serious game lets the learners experience the enjoyment of the future outcome while presenting the learning activities. While the game design aspects of serious games, such as motivational, ludic activity, or narrative elements, are relevant to create this joyful experience [16-19], it has been shown that the educational content of serious games, such as exercises, meta-cognitive, or meta-reflection support, must be adapted to the actual skill level of the learner to avoid a motivational decrease (eg, through frustration or boredom) [19-21]. To achieve these dynamic adaptations, intelligent tutoring systems have emerged to mimic distinct human tutoring interventions [22,23]. Therefore, such systems must provide two domain-specific functionalities: (1) a detailed learner analysis and, based on this analysis, (2) a recommender service for content and instructional adaptations [24] (see research question [RQ] 1 and RQ2).

A recent literature review revealed that intelligent tutoring systems combined with gamified or playful content (eg, serious games) are common in the fields of science, technology, engineering, mathematics, and language learning [22,25]. Hence, with this feasibility study, we presented a novel approach that combined an intelligent tutoring system with a serious game in the context of aural rehabilitation for adult German-speaking cochlear implant recipients (see RQ3). We wanted to encourage future researchers and developers to build more advanced computer-based auditory training by answering the following RQs:

- RQ1: How can an intelligent tutoring system estimate a cochlear implant recipient’s current level of language comprehension?
- RQ2: How can an intelligent tutoring system generate tasks for cochlear implant recipients that match their current level of language comprehension?
- RQ3: How can an intelligent tutoring system be embedded into a serious game to create adaptive and game-based auditory rehabilitation training?

### Adaptive Adjustments of Educational Systems

A digital, adaptive educational system tries to improve learning outcomes and raise engagement by altering the training application to a student’s or learner’s specific needs [21,26]. While the characteristics of these kinds of systems vary, researchers generally refer to them as computer-aided instruction, adaptive learning systems, or intelligent tutoring systems [22]. Usually, an intelligent tutoring system contains 4 conceptual models: the domain model (or content model), the learner model (or student model), the tutor model (or instructional model), and the interface model (or presentation model) [22,23,27]. The domain model contains all domain-related information pieces and their implicit and explicit structure and interdependencies [22,23,28]. The learner model captures what a person knows and does, for example, knowledge, preferred learning style, goals, or demographics [22,23,27]. The tutor model encompasses the didactic components and instructional strategy [22,23,27]. The interface model facilitates the interaction between learners and an intelligent tutoring system [22,23]. In a nutshell, the tutor model uses the learner model as the source for adaptation and the domain model and the interface model as targets for adaptation [22,23,27]. Therefore, examples of adaptation targets are specific content (eg, present feedback for specific errors), navigation (eg, the sequence of learning objects), presentation forms (eg, text vs video), and assessments (eg, difficulty level) [29].

Due to the variety of artificial intelligence systems used in educational systems in the past years [30,31], a definition of requirements for our use case is needed. First, due to the lack of available datasets about German-speaking cochlear implant recipients, a system design was needed to overcome a so-called “cold start problem” [31]. Second, due to ethical concerns, we were looking for an algorithm in the context of explainable artificial intelligence [25,30]. Regarding these preconditions, the evidence-centered design (ECD) framework seems to be a fitting methodology supporting the design and development process of the presented conceptual models.

Almond et al [32] summarized the ECD as “an approach for constructing educational assessments in terms of evidentiary arguments.” They argue that when learners fulfill tasks, they create some kind of result (work products) that incorporates (to some degree) the learner’s performance (compare with the study by Gnadlinger et al [33]). Thus, work products contain evidence about a learner’s latent competencies. Extracting evidence for competencies from performance aligns well with Forth’s [34] definition of competency as a “...set of skills and behaviors required in the performance of a task or activity within a specific context.” Hence, if a computer-based system collects this evidence, it can also model the learner’s competencies to some degree. While the ECD does not strictly depend on a specific statistical method to describe the learner model, Bayesian networks have often been used and suggested in the past [19,32]. Bayesian networks are probabilistic graphical models that hold a set of variables and their conditional dependencies as a directed acyclic graph [35]. The core idea of Bayesian networks is to use measurable variables (eg, exercise results) to infer directly immeasurable or latent variables (eg, the level of a complex competency) [35]. Hence, Bayesian networks can be used to model the learner’s competencies and continuously describe the current beliefs about these competencies by updating the measurable variables based on evidence from multiple tasks [32]. In the Conceptual Assessment Framework and Assessment Implementation section, we present how this can be achieved according to the use case of a learner model for language comprehension of cochlear implant recipients. Furthermore, we show how the other conceptual models that address these 2 prerequisites were designed and built.

### Aural Rehabilitation and Existing Auditory Training

Aural rehabilitation can be seen as a synonym for audiologic rehabilitation, auditory rehabilitation, hearing rehabilitation, and rehabilitative audiology and describes any intervention that addresses the communicative and psychosocial consequences of hearing loss [36,37]. Auditory training interventions vary in many aspects, such as training stimuli (eg, pure tones, phonemes, and complete sentences); frequency; duration of the training; and complexity [38]. It was shown that active, lexically oriented auditory training supported the learning process of adults far better than passive exercises [8]. In addition, a recent comparison of 16 studies of active auditory training provides evidence that intensive auditory and auditory-cognitive training supports the improvement of aural communication skills [38]. In a similar way this is also addressed by Deutsche Gesellschaft für Hals-Nasen-Ohren-Heilkunde, Kopf- und Hals-Chirurgie (German Society of Oto-Rhino-Laryngology, Head and Neck Surgery) [39].

Some very popular auditory training tools for English-speaking cochlear implant recipients are Angel Sound [40], the Listening Room [41], and MED-EL Academy [42]. A recent literature review of German and English computer-based auditory training shows that some auditory training systems support adaptivity and real-time feedback [9]. One prototype for a German-speaking hearing training platform was Train2hear [13,14]. It supports adaptive exercises to a certain extent and embeds different learning modules (filled with exercises) into a story about a journey through Europe. These systems generally analyze the quantity and kind of errors, the exercise duration, and the number of assistance requests to adapt exercises or training plans [13,14,40,41]. The supported adaptation of exercises can be categorized into (1) audio content—for example, differentiated by type (ie, syllables, words, sentences, and texts), similarity or complexity, and length (eg, word length); (2) hearing taxonomy—for example, differentiated into understanding, identification, discrimination, and detection; (3) exercise conditions—for example, difficulty adjustment via background noise, open or closed exercise sets, and the possibility for users to obtain assistance (eg, repetition) [13,14]. While some available platforms are well advanced according to the number of exercises and available content, none embed these exercises into a game-based learning environment. On the other hand, Garadat [43] successfully demonstrated the impact of a serious game to enhance the perceptual learning of speech by English-speaking cochlear recipients.

Hence, with this contribution, we presented a novel approach that combines an intelligent tutoring system that encountered the 2 major requirements (“cold start”—capable and explainable), with a serious game in the context of aural rehabilitation for German-speaking adult cochlear implant recipients.

## Methods

### Design and Development Process

The system design started from the perspective of assessments owing to the behavior of hearing therapists, who included exercises and tasks to assess hearing competencies and adapt the rehabilitation program. The ECD was followed because this methodology supported the design of assessments in educational systems and the inference of relevant competencies (Textbox 1).

**Main phases of the evidence-centered design framework from the study by Mislevy et al [44].**

**Domain Analysis**

“Domain analysis marshals beliefs, representations, and modes of discourse for the target domain.”

**Domain Modeling**

“Assessment developers organize insights about the domain from domain analysis... and [articulate] dependencies in knowledge, skills, and attributes in the domain, and the relationships of these capabilities to situations and activities.”

**The Conceptual Assessment Framework**

“The designers combine domain information with information about goals, constraints, and logistics to create a blueprint for an assessment.”

**Assessment Implementation**

“Assessment practitioners create functioning realizations of the models articulated in the Conceptual Assessment Framework.”

**Assessment Delivery**

“In this layer, students interact with tasks, their performances are evaluated, and feedback and reports are produced.”

### Domain Analysis and Domain Modeling Phase

**Figure 1:**
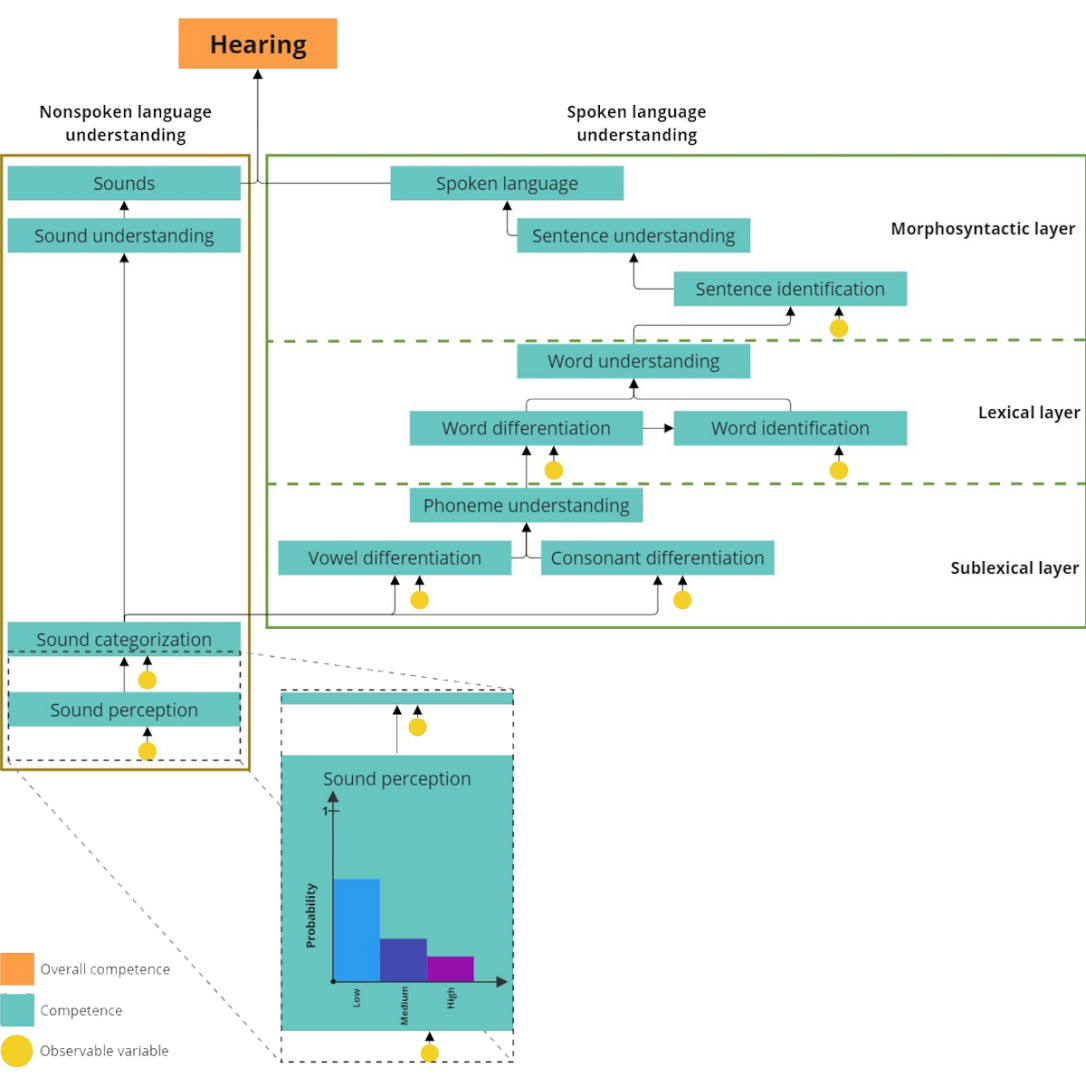
Simplified and reduced learner model for language comprehension subcompetencies.

In the beginning, an interdisciplinary team was gathered, including 2 language and speech therapists, a linguist, an audiologist, a cochlear implant surgeon, a game designer, 2 research associates with human-computer interaction backgrounds, and 2 software engineers. At the beginning of the design phase, the clinical staff gave insights into the aural rehabilitation process to the research associates. The research associates were also invited to participate in 2 face-to-face therapy sessions. Afterward, the team collaboratively designed a graphical model to describe the ability to hear and its subcompetencies in 4 distinct steps. First, the ability to hear was divided into general areas from “nonlexical language understanding” to “spoken language understanding” and additional sublayers from “nonlexical,” “lexical,” and “morphosyntactic.” Second, the experts agreed on major subcompetencies within the resulting areas, which the training environment should cover (visualized in blue in Figure 1). Third, the experts added the relationships between those subcompetencies. Finally, 7 different observable variables were defined: “sentence identification,” “word differentiation,” “word identification,” “consonant differentiation,” “vowel differentiation,” “sound categorization,” and “sound perception” (visualized as observable variables in Figure 1). The Conceptual Assessment Framework and Assessment Implementation section illustrates how these observable variables are used. The main result of this phase is a conceptional competency model pictured in Figure 1.

### Conceptual Assessment Framework and Assessment Implementation

In the 2 ECD phases explained subsequently, the conceptional definition and implementation of the 4 main models of the intelligent tutoring system and serious game were performed.

#### Learner Model

Initially, the conceptual competency model was transformed into a Bayesian network to create a machine-interpretable but human-explainable learner model. This alteration enables an ongoing use of this model for estimating the cochlear implant recipient’s current competency level in the form of competency beliefs (compare this process with Almond et al [32,45]). To do so, all nodes of this Bayesian network—the overall competence “hearing,” the subcompetencies, and observable variables visualized in Figure 1—are represented as a probability distribution of 3 states: “low,” “medium,” and “high” (compare with the node “sound perception” in Figure 1).

These random variables describe the system’s beliefs about a cochlear implant recipient’s competence—whether it is more likely to be “low,” “medium,” or “high.” In addition, all conditional probability tables within this Bayesian network were evenly distributed, which means that “phoneme understanding” depends in the same way on “vowel differentiation” as on “consonant differentiation” (Figure 1). We used these states as demonstrated in examples provided by Shute et al [19] and Almond et al [32], as they are straightforward to understand but also effectively capable of illustrating the benefit of using Bayesian networks [32].

By slightly changing the distribution of the states (“low,” “medium,” or “high”) from the observable variables according to each task result, the probability distribution will converge to a particular state over time. For example, if the cochlear implant recipient shows a feeble performance in the task type “sound perception,” the probability state “low” of the observable variable “sound perception” will increase, while the probability of “medium” and “high” will decrease (Figure 1). In addition to this described update of a specific observable variable, an inference process of the Bayesian network allows the system to update the related competencies [33]. Therefore, for example, if the cochlear implant recipient shows an excellent performance in the task type “sound perception,” the probability state “high” of the variable “sound categorization” will increase, while the probability of states “low” and “medium” will decrease. This process follows the arcs of the nodes in the hierarchy of the Bayesian network (Figure 1). By repeating this update process on individual Bayesian networks for each cochlear implant recipient according to their task results, the competence distributions of each node in this network converge to a particular state (“low,” “medium,” or “high”). In this way, an explainable learner model that can describe the beliefs of the system about the competencies of each individual cochlear implant recipient was created.

#### Domain Model and Interface Model

**Figure 2:**
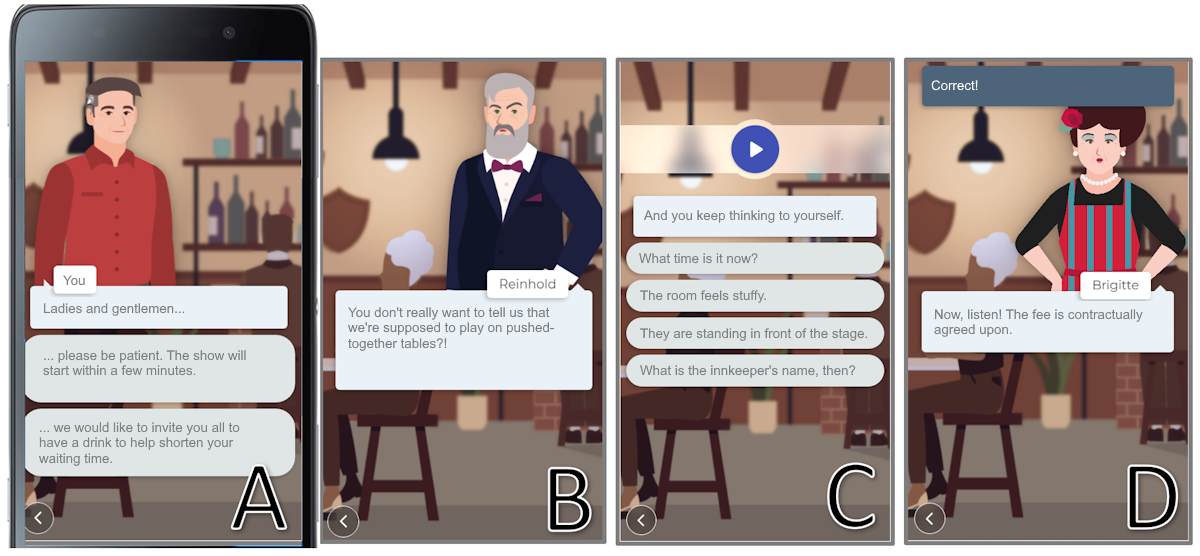
Interface of the serious game. (A) Story decision element; (B) simple dialogue element; (C) task element; and (D) feedback element at the top (notification says “Correct!”). For better readability, we translated the text into English. Note that the game is only localized in German.

The content model comprises 7 task types, each designed as single-choice tasks addressing a specific observable variable within the learner model (Figure 1). All tasks were designed and developed similarly (Figure 2C). The target group is predominantly German-speaking; therefore, only German elements are available as screenshots.

A sound is played when the task is presented and can be repeated whenever the user clicks the Play button. A question based on the task type and 2 or 4 answer options appear (Table 1). The cochlear implant recipients must select an answer to continue. After selecting an option, a notification appeared (compare with Figure 2D) indicating if the given answer was correct or incorrect. Each task type dynamically selects training items from a preconfigured pool of sound and speech assets to meet the estimated competence level of the cochlear implant recipient. The preconfigured pool holds additional (manually added and semiautomatically generated) meta-information for each sound and speech asset, which is used by the tutor model to meet the estimated competence level. To enable the tutor model to generate diverse task difficulties, 1053 audio assets with meta-information were created. Multimedia Appendix 1 provides a detailed description of the selection parameters used to determine the difficulty of a sound asset. A single default background noise was created to raise the difficulty of tasks.

**Table 1:** Single-choice quiz task types (compare with Figure 1).

Task type	Question (stem)	Options (response)
SP^a^	“Did you hear anything?”	A: “Yes”; B: “No”
SC^b^	“Does the sound fit to this category: [category]?”	A: “Yes”; B: “No”
CD^c^	“Are the two sounds the same?”	A: “Yes”; B: “No”
VD^d^	“Are the two sounds the same?”	A: “Yes”; B: “No”
WD^e^	“Are the two words the same?”	A: “Yes”; B: “No”
WI^f^	“Which word did you hear?”	A: “[word1]”; B: “[word2]”; C: “[word3]”; D: “[word4]”
SI^g^	“Which sentence did you hear?”	A: “[sent.1]”; B: “[sent. 2]”; C: “[sent. 3]”; D: “[sent. 4]”

^a^SP: sound perception.

^b^SC: sound categorization.

^c^CD: consonant differentiation.

^d^VD: vowel differentiation.

^e^WD: word differentiation.

^f^WI: word identification.

^g^SI: sentence identification.

#### Tutor Model

Whenever a task (Figure 2C) is presented to the learner, the tutor model dynamically creates it in 3 steps. The initial step involves determining the appropriate task type to choose. Our goal for this decision is to focus to some degree on the weakest competencies for rehabilitation reasons but avoid, for motivational reasons, a pure concentration on them. The decision algorithm is based on the utility theory [46]. Hence, it can be classified as a utility-based algorithm [47]. In a nutshell, such decision algorithms score all possible options and select the highest-rated one [47]. In this scenario, the system must decide between 7 different task types. The utility of each task type is calculated based on 3 criteria (Textbox 2). The criteria were weighted differently to select the task types in a nonuniform way and yet provoke a minor focus on the weaker competencies.

The second step is to generate a target task difficulty based on the estimated subcompetency level of the cochlear implant recipient, which is addressed by the selected task type (compared with Figure 1). This is achieved by converting the current probability distribution of the related observable variable from the target task type into a normalized scalar value and using this value further as a target task difficulty (eg, the target task type is “sound perception,” so the target task difficulty is calculated based on the probability distribution of the observable variable “sound perception”). The third step is to generate a task according to the task type definitions and the generated target task difficulty. For example, suppose we would like to generate a task of the task type “sound perception” with a specific target task difficulty. In that case, the algorithm must find a sound asset of type “sound” whose loudness, recurrence, and concreteness values fit the target difficulty level (compare with Table 1). Therefore, each key and distractor sound asset combination in a preconfigured task pool is a possible task. This selection algorithm for key and distractor sound assets is also based on the utility theory. Hence, the selection algorithms score all possible tasks based on the given sound asset parameters in the preconfigured task pool. The task with the closest score to the target difficulty is the best selection for the cochlear implant recipient.

As mentioned earlier, after a cochlear implant recipient completes a task, its individual Bayesian network gets updated according to the task result. In this manner, the cochlear implant recipients practice within a training loop, wherein with each new task outcome, the estimated levels of competence should progressively align more closely with the actual competence level of the recipients.

**Decision algorithm scores.**

**Task-type-repetition-score**

Higher score for task types with a low number of tasks within the last 30 tasks.

**Competence-weakness-score**

Higher score for task types that target weaker observable variables.

**Right-wrong-ratio-score**

Higher scores for task types with the lower correct or incorrect results in a row.

#### System Architecture and Serious Game Design

To practice tasks, a serious game in the form of a progressive web application was developed using an HTML5 framework to support current iOS and Android devices and their major browsers: Safari and Google Chrome. The user progress within the serious game was forwarded to a Java-based pseudonymization service (see the Data Collection section), which forwarded only the required parameters to a Node.js-based recommendation service, which implemented the described tutor model. The progressive web application contains 2 game modes: a story-driven approach (story mode) and a simple training mode (quick training mode). The story mode embeds the tasks into a graphic novel where the story is driven through dialogues and decisions (compare with Figures 2A and 2B). The story is about a group of amateur theater actresses who perform at several locations. Unfortunately, many things go wrong, so conflicts and absurd social situations happen. The player slips into the role of one of the actresses and tries to handle these conflicts between the protagonists by choosing dialogue answers wisely. There is no penalty for wrong answers. In the end, all story branches merge to a happy ending. We selected this meta-story because it allowed us to include different hearing sensation scenarios (crowded places, countryside, dialogue situations, restaurants, etc) into 1 story. The story mode contains an introductory chapter (“initial analysis”) and 9 story chapters with 15 protagonists, 12 locations, and a playtime of approximately 1 hour. Each chapter contains 10 tasks. To avoid distractions from the game flow, the preconfigured asset pools of these tasks were filled with items related to the story and dialogues at this moment. The quick training mode repurposes the existing task types and initiates a session consisting of 10 consecutive tasks, without any story content in between. Hence, only elements in type C and D in Figures 2C and 2D, respectively, were used. The main goal of this mode was to achieve as many correct answers as possible. The preconfigured pools of the quick training mode are filled with all available sound and speech assets from the story mode. After a chapter in the story mode or a quick training session is finished, the cochlear implant recipient returns to the main menu and can decide to play another chapter in the story mode or quick training session. Both modes provide feedback to the users through the responses of the protagonists, the story flow, and via an in-application notification system (compare with Figure 2D at the top of the screen). The progressive web application and all tasks support the German language exclusively.

### Assessment Delivery and Evaluation

**Figure 3:**
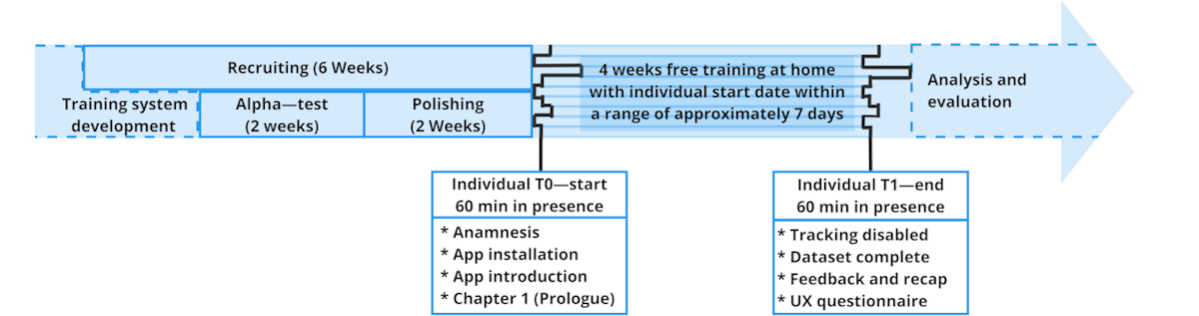
Study design inclusive timeline. UX: user experience.

The study design and evaluation approach addressed 3 main aspects: structure, process, and outcome [48]. In addition, system functionality, user perspective, and organizational context were considered [48]. Figure 3 illustrates the study timeline.

#### Ethical Considerations

Following institutional and regulatory guidelines of the University Hospital Düsseldorf, all research involving human subjects must undergo an ethics review to protect participants’ rights and welfare. This review process assessed the ethical aspects of the research, including informed consent, risk minimization, and confidentiality. Ethics approval for this project was obtained from the ethics committee at the University Hospital Düsseldorf (study number 2020-880 [49]). Before the study, all patients received detailed information regarding its objectives, methods, and possible risks. All participants provided informed consent, as required, for the primary data collection and any subsequent secondary analyses. The monitoring of the use of the progressive web app and the evaluation process were ensured to comply strictly with the European Union (EU) General Data Protection Regulation (GDPR). To ensure this, each data point was pseudonymized immediately after an automated observation was tracked. This process is described in the Data Collection section.

#### Participants

The evaluation of the intelligent tutoring system started with the recruitment of study probands. All probands are in a lifelong rehabilitation program at the Department of Otorhinolaryngology at the University Hospital Düsseldorf, covered by the German statutory health insurance. After preselection by the therapists (according to inclusion and exclusion criteria defined in the research protocol [49]), the probands were asked if they would like to participate in this study voluntarily and without valuable consideration. The team reached out to 34 persons. Due to diverse reasons (eg, lack of time, interest, and technical problems), the data of 23 cochlear implant recipients (20 women and 3 men) were collected during a training period of 4 weeks. The age of the recipients ranged from 20 to 39 years in 3 individuals; 40 to 59 years in 10 individuals; and ≥60 years in 10 individuals (mean 54, SD 14.68 y). All participants spoke German fluently (22 had German as their mother tongue). The group consisted of 35% (8/23) persons with a single-sided cochlear implant and 65% (15/23) with bilateral cochlear implant care. 39% (9/23) persons in the cohort activated their cochlear implant <4 years ago. 65% (15/23) participants said that they usually wear their cochlear implant for more than 12 hours per day, 21% (5/23) stated 9 to 12 hours per day, and 13% (3/23) between 5 and 8 hours per day. None of the participants received any other treatment during the 4 weeks. The system or the researchers did not interfere with notifications, reminders, or encouragements to motivate the participants to use the system in these 4 weeks. We conducted audiometric measurements on 22 (95%) patients at the start of the testing phase. For side-specific pure-tone audiometry, the mean value on the cochlear implant-fitted side was 32 (SD 7) dB hearing level. In the Freiburg Monosyllabic Speech Test (subset monosyllabic words), at 65 dB sound pressure level, patients correctly repeated with a mean of 53% (SD 28%) of words. At the 80 dB sound pressure level, the average was 69% (SD 24%). More details will be provided in an upcoming paper examining a clinical trial in detail.

#### Procedure

Before the 4-week training started, each cochlear implant recipient had a 60-minute appointment. At the beginning of this meeting, they installed the progressive web application on their mobile device (“bring your own device” concept), if necessary, with the help of their therapist. After a brief introduction by their therapist on how to start and interact with the progressive web application, the participants played the initial chapter, referred to as the “initial analysis.” This introductory chapter served 2 main purposes. First, the cochlear implant recipient became familiar with the application’s user interface. Second, the results of the tasks within this chapter were used to select one out of 7 preconfigured competence profiles (also known as stereotype modeling [27]). The competence profiles were defined by the clinical staff members based on their experience in hearing therapy. We assumed that a well-selected preconfigured competence profile would reduce the number of tasks a cochlear implant recipient has to solve until the system reaches a point where it properly approximates the recipient’s real competence level. As such, this addresses the so-called cold start problem [31]. This procedure also mimics an existing routine in face-to-face settings, where the therapist gets a first impression of a new patient by asking multiple basic questions. Once the first chapter was finished, the cochlear implant recipient could play each game mode (story or quick training) as much as they wanted within the following 4 weeks. After the training period, the cochlear implant recipient returned to the rehabilitation station and answered a specifically created questionnaire about the usability and user experience of the aural rehabilitation application. As a final step, their account to access the progressive web application was disabled.

The following evaluation focuses on the 4-week periods in which the participants interacted with the training system and examines the behavior of the participants and response to the training system to answer the introduced RQs.

#### Data Collection

During the 4 weeks, the task result and some meta-information (eg, timestamp and cochlear implant recipient identifier) were sent to a pseudonymization service whenever the cochlear implant recipient finished a task within the progressive web app. This service replaced the cochlear implant recipient identifier with a pseudonym and forwarded the information to the game service (an adaptation of the ADLETE Framework [33]), which was performing the update of the competency model as described in the Conceptual Assessment Framework and Assessment Implementation section. In response to the sent task result, the game service recommends the next task type and difficulty level based on the updated competency model. In the context of this study, the pseudonymization service acted as a clinical data protection layer following the EU GDPR [50].

#### Statistical Methods

Our statistical approach comprises 3 parts. The first part is a descriptive statistical analysis. It presents an overview of task results using valid percentages, mean, SD, IQR, and median. The second part is an inferential statistical analysis, which illustrates the proportions of recommended task types and error proportions among cochlear implant recipients. This includes a chi-square goodness of fit test to check for nonuniform distribution, a *Q*-*Q* plot, and a Shapiro-Wilk test for normality distribution, followed by a Kruskal-Wallis test and a Dunn post hoc test with an adjustment method, according to Holm [51], to compare distributions) to compare distributions. The last part is a generalized additive model (GAM) analysis [52]. This analysis examines the behavior of the Bayesian network for each cochlear implant recipient over time to answer RQ1 and RQ2.

Statistical significance was set at *P*<.05. We use RStudio 2023.06.1+524 “Mountain Hydrangea” developed by Posit Software, PBC for computations and visualizations and Microsoft Excel (version 2019) for some of the descriptive visualizations. Only the first 250 completed tasks were considered for comparison because only 2 participants completed more tasks (participant 1: 508; participant 2: 1024).

## Results

This section presents the results using the 3 main sections described in the Statistical Methods section: descriptive statistics, inferential statistics, and the GAM analysis. In the Discussion section, the connection between the results and RQs will be drawn.

### Descriptive Statistics

**Figure 4:**
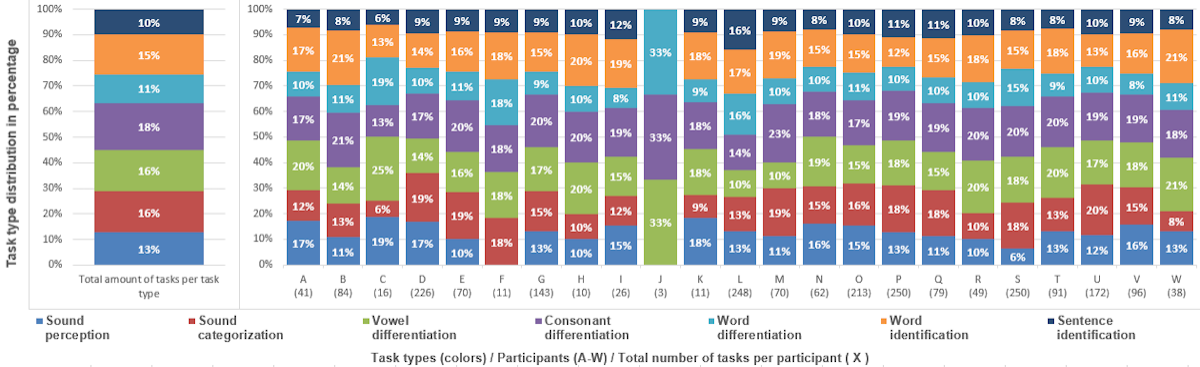
The figure shows the total number of tasks per task type and the distribution of task types as percentages for each participant. The task types are visualized in different colors. The participants are anonymized using the letters A to W.

The 23 participants completed 2259 tasks (minimum: 3, RQ1: 32, median 70, mean 98.2, RQ3: 157.50, SD 79). In total, the 23 participants solved 2004 tasks correctly and 255 tasks incorrectly (median correct/median incorrect: 65/8, arithmetic mean correct/arithmetic mean incorrect: 87.1/11.1, min correct/min incorrect: 3/0, max correct/max incorrect: 229/40). The overall arithmetic mean of the incorrect answer proportion was 9.5%. Figure 4 presents the task type distributions of each participant and allows a comparison between them. All participants received a pseudonym from A to W. The total number of fulfilled tasks is stated in round brackets below this identifier.

The system also tracked the total playtime within the 4 weeks. This can be summarized as follows: average: 159, SD 106; range 7-376 minutes.

### Inferential Statistics

**Figure 5:**
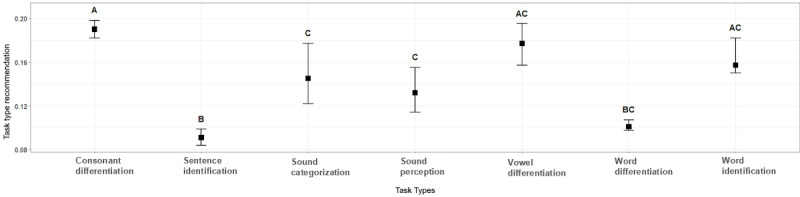
Difference between task-type recommendation proportions, including identified groups (groups A-C).

The result of the Chi-Square Goodness of Fit test (*χ*^2^_6_=86.7; *P*<.001) allowed us to reject the null hypothesis that all task types were uniformly distributed. This confirmed a first assumption that each participant’s recommended task type proportion distribution was nonuniform, as described in the Tutor Model section. To further investigate the differences between the recommended task-type proportions, we performed a Kruskal-Wallis test because we met the assumption for it by checking a *Q*-*Q* plot and performing the Shapiro-Wilk test (*P*<.001), both indicating a nonnormal distribution. The null hypothesis of the Kruskal-Wallis test could be rejected (*P*<.001), meaning that the medians of the recommended task-type proportions were different. The result of the post hoc Dunn test identified 3 groups, A, B, and C, which shared a median from a similar distribution. A compact letter display format (Figure 5) was used to visualize this. The analysis shows that the training system recommended specific task types for the participating cochlear implant recipients more likely in the following grouped order—group A: “consonant differentiation,” “vowel differentiation,” and “word identification”; followed by group C: “sound categorization” and “sound perception” and group B: “sentence identification” and “word differentiation.” The resulting analysis also indicates that the system did not recommend the task types “sentence identification” and “word differentiation” for all cochlear implant recipients in the same way, compared with “sound categorization” or “vowel differentiation,” where the variance is higher.

**Figure 6:**
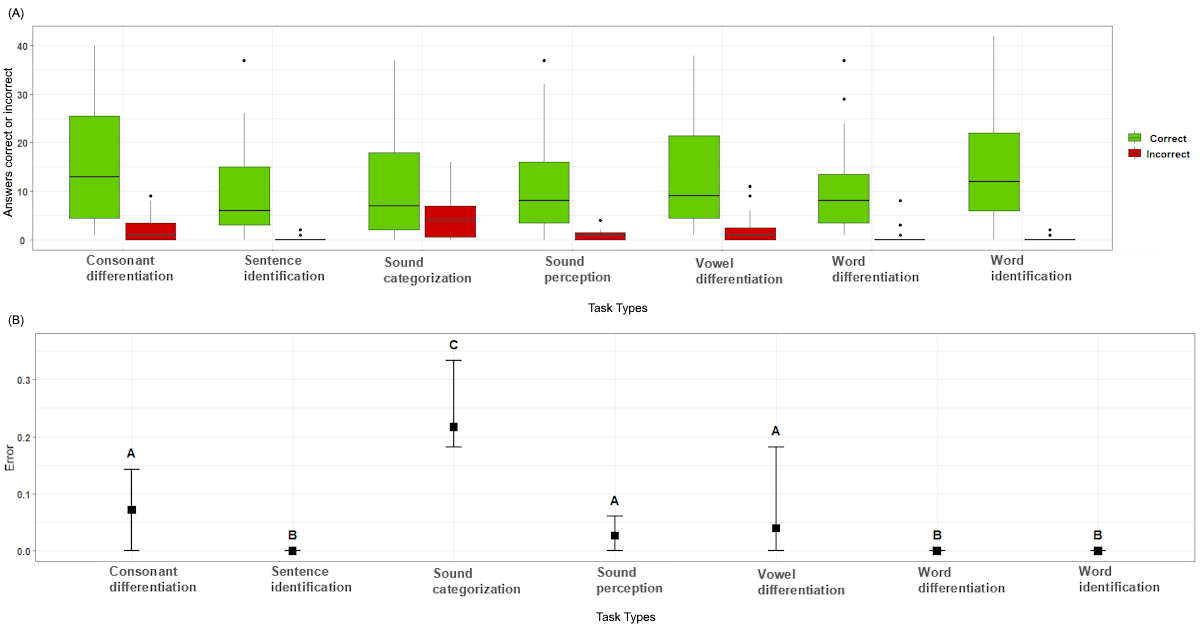
(A) Total number of tasks answered correctly or incorrectly per task type. (B) Difference between error proportion medians, including the identified groups.

Figure 6 gives an overview of the correct and incorrect answers from the recommended tasks. To further investigate the error proportions of each cochlear implant recipient based on the recommended task types, we performed a Kruskal-Wallis test because we met the assumption by checking a *Q*-*Q* plot and performing a Shapiro-Wilk test (*P*<.001) indicated a nonnormal distribution. With the result of the Kruskal-Wallis test (*P*<.001), we could reject the null hypothesis, which indicated that the medians of the error proportions from the task types were not equal. The result of the post hoc Dunn test identified 3 groups, A, B, and C, which shared a median from a similar distribution. To visualize this, a compact letter display format was used. Group C (“sound categorization”) showed the highest error proportion with a high variance between the cochlear implant recipients. Group A (“consonant differentiation,” “sound perception,” and “vowel differentiation”) showed a lower error proportion compared with group C but also showed a higher variance among the cochlear implant recipients, especially “vowel differentiation.”

### GAM Analysis

**Figure 7:**
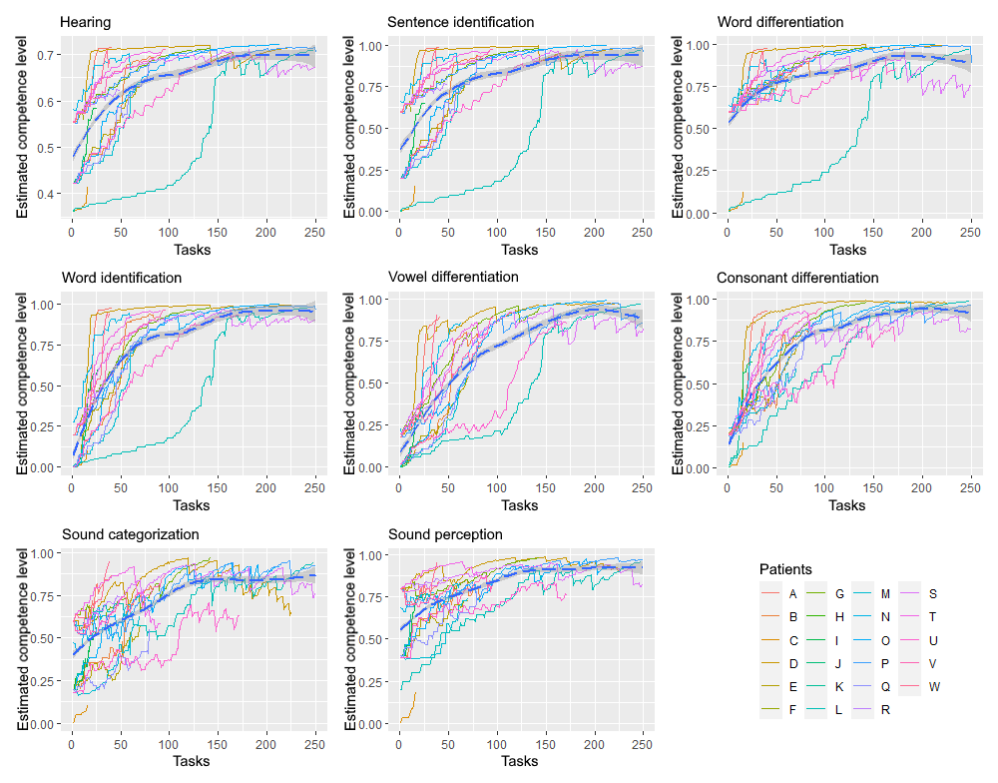
Comparing the estimated competence level of each cochlear implant recipient over time of completed tasks.

Figure 7 shows the estimated competence level over time (ie, tasks) for each cochlear implant recipient for the overall competence hearing and all observable variables (compare with Figure 2). The starting position for each competence depends on the selected initial competence profile for the cochlear implant recipient. In Figure 7, this is visible because the starting points of each plot are grouped around initial starting values. A GAM analysis [52] for each competency was performed, and the resulting model (blue dotted line) and the CI (gray area) were plotted. These plots showed that the individually updated Bayesian networks converge over time by incorporating the produced task results (evidentiary arguments) from the 7 different task types for each cochlear implant recipient. The GAM analysis indicated that the system updates the competencies quicker in the beginning (strong gradient) compared with the end (flatted curve). You can reflect on this behavior by comparing competency based on the error proportions Figure 6 with the additive model analysis Figure 7. Suppose you compare, for example, the estimated competence “sentence identification,” which has a low error rate Figure 6, with the additive model analysis of the competency “sentence identification” Figure 7. The additive model analysis of “sentence identification” showed a strong gradient in the beginning and flattened over time. Doing so for the competence “sound categorization” revealed the opposite behavior.

## Discussion

### Overview

The results were generated by 23 adult cochlear implant recipients participating in this study. The average age of the cohort was 54 years; approximately 60% of them activated their cochlear implant ≥4 years ago. Therefore, the cohort comprised experienced cochlear implant recipients. The GAM analysis of 2259 task results (including a maximum of the first 250 tasks from each participant) indicated that the system adapted to the estimated competency levels of the cochlear implant recipients more quickly in the beginning than at the end. Compared with a uniform distribution of all task types, the recommended task types differed, indicating that the system selected specific task types for each patient. This was underlined by the identified categories for the error proportion of the task types. The following discussion connects the design and development process of the game-based, adaptive auditory training with the results presented by the feasibility study to answer the 3 main RQs.

### RQ1: How Can an Intelligent Tutoring System Estimate a Cochlear Implant Recipient’s Current Level of Language Comprehension?

Following the suggestions of the ECD framework, a conceptual model that describes the language comprehension competencies and their interdependencies was designed. Our developed model aligns with previous findings like Erber’s hierarchy of listening [53] or the developed model used within the auditory training Train2hear [54].

We transformed our model into a machine- and human-interpretable probabilistic learner model (Bayesian network) to estimate the subcompetency levels of the competence language comprehension for each cochlear implant recipient. Seven single-choice task types (“sentence identification,” “word differentiation,” “word identification,” “consonant differentiation,” “vowel differentiation,” “sound categorization,” and “sound perception”) were developed that elicit a task result that incorporates evidence about the levels of the different language comprehension competencies. This evidence was used to update the learner model. This approach aligns with the suggestion from the ECD framework [32] and shows that this framework is applicable in the context of hearing rehabilitation. The developed task types empathize with already developed exercises from other similar computer-based auditory training software [14,40,54-56].

In Figure 7, we demonstrated how these Bayesian networks from the cochlear implant recipients converge over time by incorporating the produced evidence from different task types. A GAM analysis was used to visualize and reflect on the training system’s behavior. The calculated model (blue dotted line in Figure 7) indicates that the system updates the competencies quicker in the beginning (strong gradient) compared with the end (flatted curve) overall cochlear implant recipients in general. This matches with the error proportions in Figure 6 for the various task types, which also show low error proportions in general. On the one hand, this might indicate that their initial estimated competence level based on the “initial analysis” (see the Assessment Delivery section) did not fit their actual competence level. On the other hand, the generated tasks may not have met the correct difficulty level (compare further with RQ2). However, the visualization is evidence that the system adapted itself according to the behavior of the cochlear implant recipients and their competence level over time. Hence, compared with other adaptive computer-based auditory training systems [40,41,43,54,56], the illustrated use of a Bayesian network allowed us to estimate a cochlear implant recipient’s current level of language comprehension based on the required subcompetencies and their interdependencies.

### RQ2: How Can an Intelligent Tutoring System Generate Tasks for Cochlear Implant Recipients That Match Their Current Level of Language Comprehension?

The definition of task types serves as a blueprint to generate tasks. The task types were designed to use the individual estimated competence level to select the appropriate target key and distractor options from sound asset pools with a utility-based algorithm. To examine the system behavior, we analyzed each cochlear implant recipient’s first 250 fulfilled tasks and saw a strong variation between the fulfilled tasks (SD 79). However, the median of 70 indicates that 50% of all participants finished ≥70 tasks. These results and the total playtime (mean 159 min) already gave some evidence that cochlear implant recipients were willing to interact for a longer period with such an application. This finding is congruent with the results in the study by Völter et al [54], which indicated a high adherence rate for adaptive computer-based auditory training systems. We would like to examine this and the reason for their behavior in detail in an upcoming publication.

Our analysis of the recommendation system showed that the chosen task types were not uniformly distributed. Our deeper investigation revealed that “consonant differentiation,” “vowel differentiation,” and “word identification” were more likely chosen for the cochlear implant recipients (Figure 5). Furthermore, the examined error proportions associated with the 7 task types could be categorized into 3 groups (Figure 6). These identified categories do not align with the discovered categories from the recommended task-type proportions (Figure 5), which emphasizes the selection algorithm to consider different parameters rather than solely relying on correct or incorrect input. Figure 6 also indicates that the exercises were too easy even when they became more difficult because the error proportions were very low. Furthermore, there is a difference in the error proportion of the task types, which indicates that certain types (mainly “sound categorization”) seem more difficult to answer correctly. There might be various reasons for this result. For example, the task type “sound categorization” might have been misinterpreted by the target group, or the used sound assets did not meet the actual competence level. Therefore, further investigation of the task types and their difficulties would be a valid next research step.

### RQ3: How Can an Intelligent Tutoring System Be Embedded Into a Serious Game to Create an Adaptive and Game-Based Auditory Rehabilitation Training?

We provided a detailed explanation of the conceptual and technological approach for building an intelligent tutoring system for aural rehabilitation, adhering to the recommendations for defining the 4 main models of an intelligent tutoring system: the domain model, learner model, tutor model, and interface model [22]. The intelligent tutoring system was designed to overcome two major requirements: (1) the so-called “cold start problem” due to the lack of available data in advance [31] and (2) the “explainable intelligence” due to ethical concerns in the context of eHealth [25]. The cold start problem was addressed using a Bayesian network as a learner model, designed based on expert knowledge, so initial learner data were unnecessary. In addition, the system can be explained by visualizing a Bayesian network and its local conditional probability distributions over time (Figure 7). This allows us to model each individual cochlear implant recipient based on their input, which stands in contrast to current common machine learning approaches that try to find one categorization model based on available data of a whole cohort (compare, eg, with the studies by Leduc-McNiven et al [57] and Goumopoulos et al [58]).

Furthermore, this description thoroughly shows how to embed this intelligent tutoring system via a task system into a serious game. The developed serious game in the form of a progressive web application supports 2 different game modes. The story mode embeds the tasks into a graphic novel–like game environment, while the quick training mode allows cochlear implant participants to practice 10 tasks in a row in a training-like environment. Hence, from a software architectural point of view, an autonomously functioning task system enables the generation of diverse scenarios by reusing identical task types. With the dialogue-driven story mode, we followed the initially stated argument that a serious game should allow learners to experience the enjoyment of the future outcome of their learning process [15]. Here, we allow the cochlear implant recipient to experience participation in conversations even in difficult situations (eg, at a restaurant, compare with parts A, B, C, and D in Figure 2). Arguably, the story mode might miss key elements that would classify it as a serious game. However, we emphasize the suggestion of the G, P, and S serious game classification to distinguish between serious games with a game-based gameplay component (strong goal oriented and rule based) and a play-based one (indirectly measurable goals) [59] and consider our serious game as a play-based one. It would be interesting to examine the motivation and flow experience of the cochlear implant recipients of serious games following a more play-based gameplay approach in further studies.

Unlike existing training platforms, which primarily analyze the quantity and type of errors [13,14,40,41,54] (as detailed in the Aural Rehabilitation and Existing Auditory Training section), our approach allows us to use the learning trajectory of each hearing competency and their interdependencies to recommend the next suitable training task for a cochlear implant recipient. By presenting a way to integrate such a training system into a serious game, we address the call from a recent literature review on the personalization of serious games to explore methods of incorporating learning progress into intelligent tutoring systems rather than relying solely on task results [60].

### Limitations

First, compared with studies in educational science where the ECD framework originated, the total number of participants in this study was relatively low because of the available funding for the conducted feasibility study. Hence, cautious generalization of the results is required. Nevertheless, the results presented in this publication are a valuable base for a formal sample size calculation (eg, Cochran’s sample size formula) for future studies. Second, only cochlear implant recipients from the Department of Otorhinolaryngology at the University Hospital Düsseldorf volunteered in this study, raising the possibility of an existing self-selection bias. This bias might also include side effects from different types of cochlear implant sound processors used by the cochlear implant recipients. Finally, we did not evaluate the competencies of the participating cochlear implant recipients with another measurement instrument due to the lack of standardized tests for the specific subcompetencies. The study was conducted to answer specific RQs and comply strictly with the EU GDPR. Hence, further post hoc analysis can only be applied to the specific tracked variables defined at the beginning of the project. Therefore, for example, questions regarding the preferred game mode of the cochlear implant recipients must be answered in future studies.

### Future Work

While the presented results allow a system-wise interpretation of the cochlear implant recipient’s input, future studies should focus on observing the cochlear implant recipient’s use behavior. This also includes the responses of cochlear implant recipients to specific game mechanics (eg, the preferred game mode) or interaction time. In addition, since the results indicated that the given tasks seemed to be easily solvable by the participating cochlear implant recipients, further evaluation is required to find out if the selected evidentiary arguments of the developed task types hold in general for cochlear implant recipients. In addition, examining the long-term effects of adaptive and game-based auditory training in the rehabilitation process of cochlear implant recipients versus traditional rehabilitation techniques would be very valuable.

For further research from a technological perspective, it might be interesting to investigate ways to use the potential of Bayesian networks to create synthetic data, as [25] suggested via agent-based simulations [58,60] for early development stages. Because the defined Bayesian network describes a competency ontology, future developments might consider coupling them with game mechanic ontologies illustrated, for example, in the study by Goumopoulos and Igoumenakis [61]. Such a coupling might be possible using generative artificial intelligence (eg, [62]) for immediate on-demand generation of training tasks that meet task difficulty and game mechanic requirements. Hence, a personalized recommendation approach similar to the one presented in this paper might lead to prompt generators that will reduce the cost of training content creation significantly.

Besides these technological perspectives, we already see a shortage of specialized therapists and trainers supporting adult cochlear implant recipients in Germany (compare with the study by Völter et al [54]). With the foreseeable increase in the number of adult individuals with cochlear implants, new ways of hearing rehabilitation are needed to address this growing gap between both groups.

### Conclusions

This is the first attempt to map cochlear implant recipients’ language comprehension competencies using a Bayesian learner model for an intelligent tutoring system in cochlear implant rehabilitation. We integrated this system into an adaptive, game-based auditory rehabilitation training, addressing the need for an explainable design and solving the cold start problem owing to the lack of initial user data. A feasibility study with 23 cochlear implant recipients showed that the system adapts to the estimated users’ competency levels. The task system tailored tasks to each patient, as indicated by comparing error proportions and GAM analysis. With this work, we contribute and support the future game-based designs of computer-based, intelligent cochlear implant rehabilitation therapies; general hearing therapies; and similar fields in the eHealth context, where the personalized adaptation of a training environment is required to meet the needs of individuals.

## Appendix

Multimedia Appendix 1 Extended versions of some of the published figures and tables.

## Abbreviations

ECD: evidence-centered design
EU: European Union
GAM: generalized additive model
GDPR: General Data Protection Regulation
RQ: research question

## References

[1] (2021). World report on hearing. World Health Organization (WHO) Global Reports.

[2] Elflein J (2019). Number of cases of hearing loss by severity worldwide. Statista.

[3] Távora-Vieira D, Marino R (2019). Re-training the deaf ear: auditory training for adult cochlear implant users with singlesided deafness. Cochlear Implants Int.

[4] Frijns JH, Briaire JJ, Jaeger D, Jung R (2015). Auditory prosthesis. Encyclopedia of Computational Neuroscience.

[5] Stöver T, Plontke SK, Guntinas-Lichius O, Welkoborsky H, Zahnert T, Delank KW, Deitmer T, Esser D, Dietz A, Wienke A, Loth A, Dazert S (2023). Konzeption und Implementierung eines Zertifizierungssystems zur Qualitätssicherung der Cochlea-Implantat-Versorgung in Deutschland. HNO.

[6] Olson AD (2015). Options for auditory training for adults with hearing loss. Semin Hear.

[7] (2021). Cochlea implants: fact sheet - hearing and balance. National Institute on Deafness and other Communication Disorders (NIDCD).

[8] Drouin JR, Theodore RM (2020). Leveraging interdisciplinary perspectives to optimize auditory training for cochlear implant users. Lang Linguist Compass.

[9] Völter C, Schirmer C, Stöckmann C, Dazert S (2020). Computerbasiertes Hörtraining in der Hörrehabilitation Erwachsener nach Cochleaimplantation. HNO.

[10] Dornhoffer JR, Reddy P, Ma C, Schvartz-Leyzac KC, Dubno JR, McRackan TR (2022). Use of auditory training and its influence on early cochlear implant outcomes in adults. Otol Neurotol.

[11] Ratnanather JT, Bhattacharya R, Heston MB, Song J, Fernandez LR, Lim HS, Lee S, Tam E, Yoo S, Bae S, Lam I, Jeon HW, Chang SA, Koo J (2021). An mHealth app (Speech Banana) for auditory training: app design and development study. JMIR Mhealth Uhealth.

[12] Stacey PC, Raine CH, O'Donoghue GM, Tapper L, Twomey T, Summerfield AQ (2010). Effectiveness of computer-based auditory training for adult users of cochlear implants. Int J Audiol.

[13] Völter C, Schirmer C, Röber M, Hinsen D, Dazert S, Bilda K (2021). Neue Wege in der Hörrehabilitation nach Cochleaimplantation. HNO.

[14] Völter C, Schirmer C, Hinsen D, Roeber M, Dazert S, Bilda K (2020). Therapist-guided telerehabilitation for adult cochlear implant users: developmental and feasibility study. JMIR Rehabil Assist Technol.

[15] Drummond D, Hadchouel A, Tesnière A (2017). Serious games for health: three steps forwards. Adv Simul (Lond).

[16] Mildner P, Mueller FF, Dörner R, Göbel S, Effelsberg W, Wiemeyer J (2016). Design of serious games. Serious Games: Foundations, Concepts and Practice.

[17] Peirce N, Conlan O, Wade V (2008). Adaptive educational games: providing non-invasive personalised learning experiences. Proceedings of the 2008 Second IEEE International Conference on Digital Game and Intelligent Toy Enhanced Learning.

[18] Shute VJ, Leighton JP, Jang EE, Chu MW (2016). Advances in the science of assessment. Educ Assess.

[19] Shute VJ, Rahimi S, Smith G, Ke F, Almond R, Dai CP, Kuba R, Liu Z, Yang X, Sun C (2020). Maximizing learning without sacrificing the fun: stealth assessment, adaptivity and learning supports in educational games. Comput Assist Learn.

[20] Lach E (2017). Dynamic difficulty adjustment for serious game using modified evolutionary algorithm. Proceedings of the 16th International Conference on Artificial Intelligence and Soft Computing.

[21] Vanbecelaere S, Van den Berghe K, Cornillie F, Sasanguie D, Reynvoet B, Depaepe F (2019). The effectiveness of adaptive versus non‐adaptive learning with digital educational games. Comput Assist Learn.

[22] Ramadhan A, Warnars HL, Razak FH (2023). Combining intelligent tutoring systems and gamification: a systematic literature review. Educ Inf Technol.

[23] Wang H, Tlili A, Huang R, Cai Z, Li M, Cheng Z, Yang D, Li M, Zhu X, Fei C (2023). Examining the applications of intelligent tutoring systems in real educational contexts: a systematic literature review from the social experiment perspective. Educ Inf Technol (Dordr).

[24] Shute VJ, Zapata‐Rivera D (2007). Adaptive technologies. ETS Research Report Series.

[25] Pérez J, Castro M, López G (2023). Serious games and AI: challenges and opportunities for computational social science. IEEE Access.

[26] Gao Y (2023). The potential of adaptive learning systems to enhance learning outcomes: a meta-analysis. Department of Educational Psychology, University of Alberta.

[27] Vandewaetere M, Desmet P, Clarebout G (2011). The contribution of learner characteristics in the development of computer-based adaptive learning environments. Comput Human Behav.

[28] Shute V, Towle B (2003). Adaptive e-learning. Educ Psychol.

[29] Martin F, Chen Y, Moore RL, Westine CD (2020). Systematic review of adaptive learning research designs, context, strategies, and technologies from 2009 to 2018. Education Tech Research Dev.

[30] Hur P, Lee H, Bhat S, Bosch N (2022). Using machine learning explainability methods to personalize interventions for students. Proceedings of the 15th International Conference on Educational Data Mining.

[31] Ricci F, Rokach L, Shapira B (2015). Recommender Systems Handbook.

[32] Almond RG, Mislevy RJ, Steinberg LS, Yan D, Williamson DM (2015). Bayesian Networks in Educational Assessment (Statistics for Social and Behavioral Sciences).

[33] Gnadlinger F, Selmanagić A, Simbeck K, Kriglstein S (2023). Adapting is difficult. Introducing a generic adaptive learning framework for learner modeling and task recommendation based on dynamic Bayesian networks. Proceedings of the 15th International Conference on Computer Supported Education.

[34] Forth S (2021). What are skills? What are competencies? An update on the IEEE 1484.20.2 work on defining competencies. Ibbaka.

[35] Uglanova I (2021). Model criticism of bayesian networks in educational assessment: a systematic review. Pract Assess Res Eval.

[36] Aural rehabilitation for adults. American Speech-Language-Hearing Association.

[37] Montano JJ, Spitzer JB (2021). Adult Audiologic Rehabilitation. 3rd edition.

[38] Stropahl M, Besser J, Launer S (2020). Auditory training supports auditory rehabilitation: a state-of-the-art review. Ear Hear.

[39] Präsidium der DGHNO (2018). Empfehlungen zur Struktur, Organisation, Ausstattung, Qualifikation und Qualitätssicherung in der Versorgung von Patienten mit einem Cochlea-Implantat in der Bundesrepublik Deutschland. Deutsche Gesellschaft für Hals-Nasen-Ohren-Heilkunde, Kopf- und Hals-Chirurgie (DGHNO).

[40] (2012). Angel SoundTM. Emily Fu Foundation.

[41] (2023). The listening room. Advanced Bionics AG and Affiliates.

[42] (2023). MED-EL Academy: learning platform on hearing solutions. myMED-EL - Academy.

[43] Garadat SN (2023). Development and beta testing of serious game-based auditory training application to enhance perceptual learning of speech in cochlear implant recipients. Am J Audiol.

[44] Mislevy RJ, Behrens JT, Dicerbo KE, Levy R (2012). Design and discovery in educational assessment: evidence-centered design, psychometrics, and educational data mining. J Educ Data Min.

[45] Almond RG, Mulder J, Hemat LA, Yan D (2006). Bayesian network models for local dependence among observable outcome variables. ETS Res Rep Ser.

[46] Fishburn P, Gass SI, Fu MC (2001). Utility theory. Encyclopedia of Operations Research and Management Science.

[47] Graham D, Rabin S (2019). An introduction to utility theory. Game AI Pro 360: Guide to Architecture.

[48] Cornford T, Doukidis G, Forster D (1994). Experience with a structure, process and outcome framework for evaluating an information system. Omega.

[49] Klenzner T, Schatton D, Werminghaus M, Jutta RG (2020). ProWear cochlea - development, testing and evaluation of a hearing training program for cochlear implant patients. The Federal Institute for Drugs and Medical Devices.

[50] (2016). Regulation (EU) 2016/679 of the European Parliament and of the Council. European Parliament, Council of the European Union.

[51] Mangiafico SS (2023). Summary and analysis of extension program evaluation in R, version 1.20.07. R Companion.

[52] Park JY, Kim J, Debeer D, Van den Noortgate W (2021). Generalized additive modeling for learning trajectories in e-learning environments. Proceedings of the 85th Annual Conference of the Psychometric Society on Quantitative Psychology.

[53] Erber NP (1982). Auditory Training.

[54] Völter C, Stöckmann C, Schirmer C, Dazert S (2021). Tablet-based telerehabilitation versus conventional face-to-face rehabilitation after cochlear implantation: prospective intervention pilot study. JMIR Rehabil Assist Technol.

[55] Tuz D, Isikhan SY, Yücel E (2021). Developing the computer-based auditory training program for adults with hearing impairment. Med Biol Eng Comput.

[56] Reynard P, Attina V, Idriss S, Hermann R, Barilly C, Veuillet E, Joly CA, Thai-Van H (2022). Effect of serious gaming on speech-in-noise intelligibility in adult cochlear implantees: a randomized controlled study. J Clin Med.

[57] Leduc-McNiven K, Dion RT, Mukhi SN, McLeod RD, Friesen MR (2018). Machine learning and serious games: opportunities and requirements for detection of mild cognitive impairment. J Med Artif Intell.

[58] Goumopoulos C, Skikos G, Karapapas C, Frounta M, Koumanakos G (2021). Applying serious games and machine learning for cognitive training and screening: the COGNIPLAT approach. Proceedings of the 25th Pan-Hellenic Conference on Informatics.

[59] Djaouti D, Alvarez J, Jessel JP, Felicia P (2011). Classifying serious games: the G/P/S model. Handbook of Research on Improving Learning and Motivation through Educational Games: Multidisciplinary Approaches.

[60] Pérez J, Castro M, Awad E, López G (2024). Generation of probabilistic synthetic data for serious games: a case study on cyberbullying. Knowl Based Syst.

[61] Goumopoulos C, Igoumenakis I (2020). An ontology based game platform for mild cognitive impairment rehabilitation. Proceedings of the 6th International Conference on Information and Communication Technologies for Ageing Well and e-Health.

[62] Zeng Y, Tang Q, Chen S, Chen X (2024). Integration of a large language model with augmentative and alternative communication tool for oncological aphasia rehabilitation. Asia Pac J Oncol Nurs.

