# Supplementary material for: Incorporating an Intelligent Tutoring System Into a Game-Based Auditory Rehabilitation Training for Adult Cochlear Implant Recipients: Algorithm Development and Validation
Source: JMIR Serious Games. 2024 Dec 3;12:e55231. doi: 10.2196/55231 (PMC11653422; doi:10.2196/55231)

**Appendix**

Table a-1: Single choice quiz task types for non-spoken and spoken language comprehension (see Figure 1)

| Task Type | Question  (Stem) | Options  (Response) | Key and Distractor Sound Asset(s) +  Selection Parameter To Describe the Task Difficulty |
| --- | --- | --- | --- |
|  |  |  |  |
| SP^a^ | “Did you hear anything?” | A: "Yes" B: "No" | A key sound asset is selected from a pre-configured pool based on the meta-information: type=”Sound”, Loudness, Recurrence, and Concreteness. |
| SC^b^ | “Does the sound fit to this category: [category]?” | A: "Yes" B: "No" | A key sound asset is selected from a pre-configured pool based on the meta-information: type="Sound", Concreteness, Loudness, Recurrence, Category, and Category Prototype Proximity. |
| CD^c^ | “Are the two sounds the same?” | A: "Yes" B: "No" | A key and a distractor consonant sound asset are selected from a pre-configured pool based on the meta-information: type=”Consonant”,  ConsonantClass [Plosive\| Fricative\| Nasal], ConsonantLength, and Voicing [voiced\| voiceless]. |
| VD^d^ | “Are the two sounds the same?” | A: "Yes" B: "No" | A key and a distractor consonant sound asset are selected from a pre-configured pool based on the meta-information: type=”Consonant”,  ConsonantClass [Plosive\| Fricative\| Nasal], ConsonantLength and Voicing [voiced\| voiceless]. |
| WD^e^ | “Are the two words the same?” | A: "Yes" B: "No" | A key and a distractor word sound asset are selected from a pre-configured pool based on the meta-information: type=”Word”, WordTranscript, PhoneticTranscription -IPA alphabet transcription, PhonemeCount, SyllableCount, Frequency, and Compound (=the process of word formation that creates compound lexemes). |

| Task Type | Question  (Stem) | Options  (Response) | Key and Distractor Sound Asset(s) +  Selection Parameter To Describe the Task Difficulty |
| --- | --- | --- | --- |
|  |  |  |  |
| WI^f^ | “Which word did you hear?” | A: "[word1]" B: "[word2]" C: "[word3]" D: "[word4]" | A key word sound asset and three distractor word sound assets are selected from a pre-configured pool based on the meta-information: type=”Word”, WordTranscript, Phonetic-Transcription string - IPA alphabet transcription, Phoneme-Count, SyllableCount, and Frequency. |
| SI^g^ | “Which sentence did you hear?” | A: "[sent.1]" B: "[sent. 2]" C: "[sent. 3]" D: "[sent. 4]" | A key and three distractor sentence sound asset are selected from a pre-configured pool based on the meta-information: type=”Sentence”, SentenceTranscript, WordCount, RecordType [declarative\| question\| prompt], VerbValency [transitive\| ditransitive\| intransitive],  SentenceStructure [light\| middle\| heavy ],  GramaticalTense [present\| past\| future ],  Mode [indicative \| conditional], GenusVerbi [active\| passive], and NeighborhoodSize – the number of similar words between both sentences. |

^a^ Sound Perception, ^b^ Sound Categorization, ^c^ Consonant Differentiation,
^d^ Vowel Differentiation, ^e^ Word Differentiation, ^f^ Word Identification,
^g^ Sentence Identification

Figure a-1: Within the manuscript, we translated the text of the following figure into English for better readability. The following figure shows the original text in German.


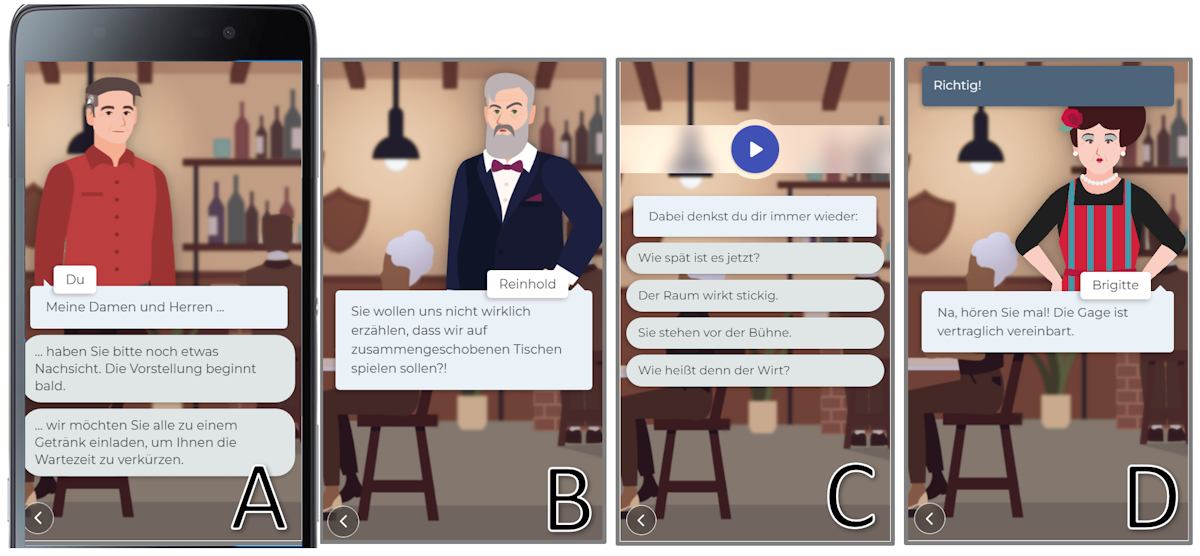

Supplement: Multimedia Appendix 1 [file games_v12i1e55231_app1.docx]
